# A theoretical design method for the nozzle pitch of air-flotation system to reduce large liquid crystal display glass substrate’s deformation

**DOI:** 10.1038/s41598-020-76793-w

**Published:** 2020-11-16

**Authors:** Bin Huang, Teng Fu, Xiao-bo Xu, Mei-xian Chen, Yang Zhou, Yong Zhang, Shan-lin Liu

**Affiliations:** Institution of Instrument Science and Opto-Electronics Engineering, Hefei University of Technology, Hefei, China

**Keywords:** Design, synthesis and processing, Mechanical engineering

## Abstract

The deformation of large glass substrate in air-flotation system affects detection accuracy of inspection instrument. According to the gas lubrication theory, Timoshenko’s thin film theory and the simulation figure of the pressure distribution of the air film flow field, the air load distribution model of the air film is established, and the deformation expression of the large liquid crystal glass substrate in air-flotation system is given. On this basis, a theoretical design method for designing nozzle pitch of orifice throttling air-flotation system was proposed. Combined with examples, the results of theories and simulation are compared. An experiment on the deformation of the glass substrate was carried out on an experimental prototype. The difference between the experimental results and the theoretical results does not exceed 10%.

## Introduction

One of the most important inspection items for the quality inspection of large liquid crystal glass substrates is the detection of surface and internal defects. The inspection instrument is required to not only identify the type and size of the defects, but also determine the location of the glass substrate. Air flotation transmission possesses the advantages of stable transmission, no pollution, no contact scratches, etc. It is the mainstream choice of support and transportation technology in an optical automatic detection instrument for defects of large liquid crystal display glass substrates^1^.

**Figure 1:**
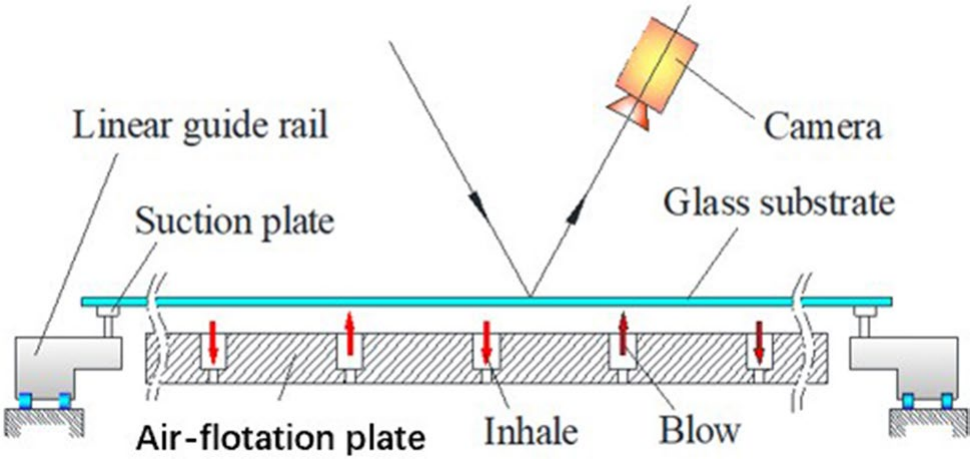
Principle of air-flotation transmission.

In the optical automatic detection instrument for defects of glass substrate, the principle of the air flotation is shown in Fig. 1: The air-flotation plate is provided with positive pressured air injection holes and negative pressured vacuum holes, which are arranged in array. The air ejected by the air injection holes forms a supporting air film between the glass and the air-flotation plate, and the function of the vacuum hole is to improve the stability of the air film. In the inspection process, the guide rail drives a glass substrate to move with the help of suction cups. The camera scans the glass and determines the location and type of defects after image processing.

The large glass substrate will be deformed on the air-flotation plate due to the uneven pressure distribution of the air float flow field in the gap between the glass substrate and the air-flotation plate, which is an important factor that interferes with the detection accuracy of optical instruments. The nozzle pitch (air hole spacing) directly affect the deformation of the glass substrate, so the proper design of the nozzle pitch is the key to controlling the glass substrate deformation amount not to exceed the allowable value.

The deformation of the glass substrate in the porous air-flotation system has attracted the attention of researchers. Amano et al.^2^ developed a new non-contact transport system based on the combination of porous positive and negative pressures and analyzed the pressure distribution of air film based on Reynolds equation; Lee et al.^3^ established a gas flow model in the porous surface gap and calculated the pressure distribution of the air film and the relative deformation of the glass substrate; Miyatake et al.^4^ studied the deformation of glass substrate during transmission process through simulation and experiment.

Orifice throttle is also one of the common throttling methods for air flotation. Compared with the porous air flotation system, the pressure distribution of the air film in the orifice throttling air-flotation system is more uneven. For example, Chandra et al.^5^ used the finite element method to study the pressure distribution of the air film, and the results showed that the pressure near the air hole has increased; Zhong Wei et al.^6^ compared the glass deformation under the conditions of porous throttling and orifice throttling through experiments. However, the orifice throttling air-flotation system is low in cost and easy to maintain and use. By appropriately designing the nozzle pitch, the deformation of the glass substrate can be controlled within the allowable range, which has a large engineering application value in the design of large glass substrate inspection instruments.

There are few studies on the deformation of the glass substrate in the orifice throttling air-flotation system. In the engineering design, the determination of the nozzle pitch is inseparable from engineering experience. In this paper, by studying the air load distribution model and the deformation of the glass substrate under the support of air flotation, the calculation method of designing the nozzle pitch in the air- flotation system is given.

## Methods

### Force analysis of glass substrate

**Figure 2:**
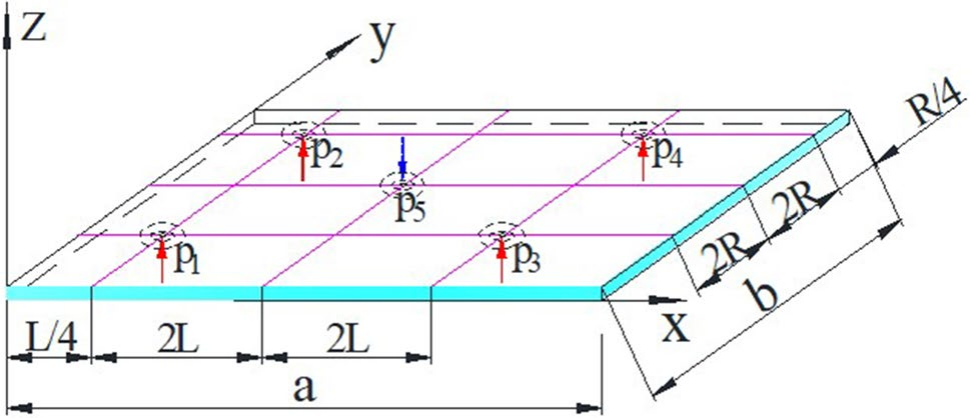
The load on the glass substrate unit.

In the air-flotation system with air holes arranged in a rectangular array, the glass substrate is subjected to three forces (take one unit as an example), as shown in Fig. 2: p1, p2, p3, p4 represent upward air flotation force, and p5 represents the suction force in the downward direction, in addition, the gravity G of the glass plate itself (not shown in the figure) is also in the downward direction. The glass substrate is in equilibrium under the above three forces.

**Figure 3:**
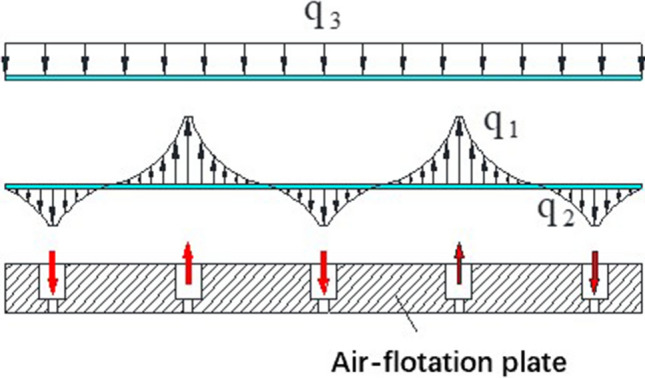
Cross-section of the air-flotation plate.

From the cross-section of the air-flotation support plate shown in Fig. 3, the glass substrate is subjected to three kinds of forces in the air-flotation system: upward air-flotation force, its load is represented by q1; downward suction force, its load is represented by q2; downward gravity, Its load is represented by q3. q3 is the weight per unit area of the glass substrate, and q3 is a constant.

### Air load on the glass substrate

**Figure 4:**
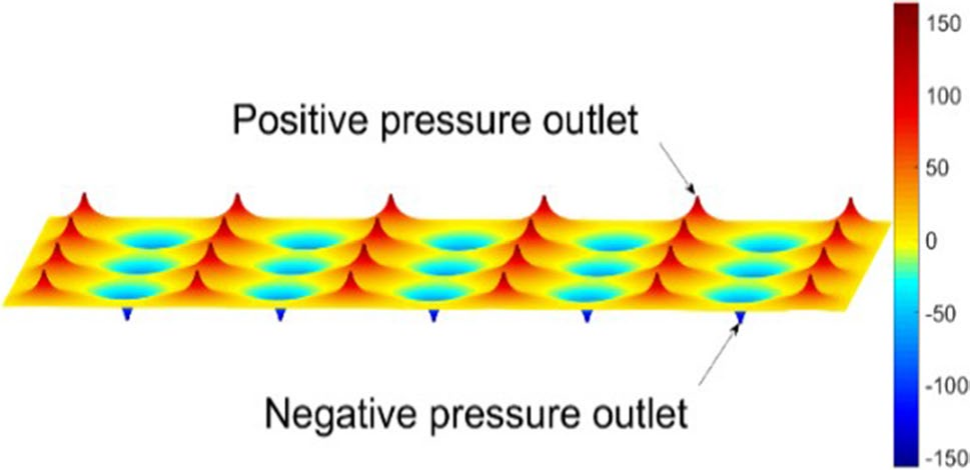
Diagram of air film pressure distribution.

The air-flotation system with air holes arranged in a rectangular array, it can be discovered from the air film pressure distribution simulation diagram (Fig. 4) that the air pressure on the circumference centered on the air holes is almost equal. Therefore, to determine the pressure distribution near the air hole, it can be assumed that the opposite surface of the air-flotation plate and the glass substrate is circular, and the air loads q1 and q2 acting on the glass substrate can be obtained according to the gas lubrication theory.

**Figure 5:**
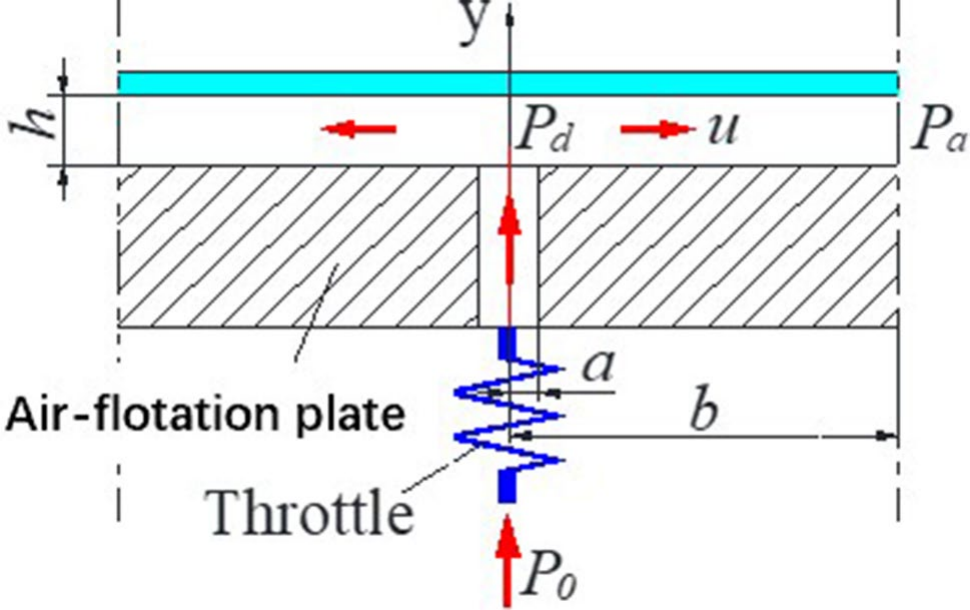
Air-flotation support model.

In the air-flotation support model shown in Fig. 5: the supply system sends air with a pressure of p_0_ to the supply hole on the air-flotation plate through the throttle, and p_d_ is the air pressure at the outlet of the hole. p_a_ is the pressure at the junction of the supply and vacuum pressure area in the air film (its value is equal to atmospheric pressure). a is the radius of the pressure chamber. b is the distance from the center of the hole to the junction of the supply and vacuum pressure area in the air film, and h is the thickness of the air film. The velocity of the air-flow flowing radially outward is u, and the viscosity of the air is μ. Because the air film pressure required to float the glass substrate is very small and the air flow velocity is low, the following simplified Navier–Stokes equation^7^ can be used to describe the air-flow between the air-flotation plate and the glass substrate:

1 $$ \frac{{\partial^{2} {\text{u}}}}{{\partial {\text{y}}^{2} }} = \frac{1}{\upmu }\frac{{\partial {\text{p}}}}{{\partial {\text{r}}}} $$

where r is a certain radius from the center of the hole, and its value is between a and b.

Integrate formula (1) and consider the boundary conditions: when y = 0 and y = h, u = 0, then

2 $$ {\text{u}} = \frac{1}{2\upmu }\frac{{d{\text{p}}}}{{d{\text{r}}}}{\text{y}}\left( {{\text{y}} - {\text{h}}} \right) $$

The mass flow through the annulus section with height h and radius r is

3 $$ {\text{q}}_{{\text{m}}} = 2\uppi {\text{r}}\uprho \mathop \smallint \limits_{0}^{{\text{h}}} {\text{udy}} $$

Combined Eqs. (2) and (3), Eq. (4) can be obtained:

4 $$ \frac{{{\text{dp}}}}{{{\text{dr}}}} = \mp \frac{{6\upmu {\text{q}}_{{\text{m}}} }}{{\uppi {\text{r}}\uprho {\text{h}}^{3} }} $$

In the Eq. (4), the negative sign applies to the air film flow field near the air supply hole, indicating that the air film pressure decreases in the direction of increasing r; the positive sign applies to the air film flow field near the vacuum hole, indicating the air film pressure increases along the direction of increasing r.

Assuming that the gas is incompressible, $\uprho$ in Eq. (4) can be determined as a constant.

Since p = p_d_ at r = a in the air film, the pressure p at any radius r (a ≦ r ≦ b) can be obtained by the integral operation of Eq. (4):

5 $$ {\text{p}} - {\text{p}}_{{\text{d}}} = \mp \frac{{6\upmu {\text{q}}_{{\text{m}}} }}{{\uppi \uprho {\text{h}}^{3} }}\ln \left( {\frac{{\text{r}}}{{\text{a}}}} \right) $$

In Eq. (5), make r = b, and note that p = p_a_ at r = b in the air film, then:

6 $$ {\text{p}}_{{\text{a}}} - {\text{p}}_{{\text{d}}} = \mp \frac{{6\upmu {\text{q}}_{{\text{m}}} }}{{\uppi \uprho {\text{h}}^{3} }}\ln \left( {\frac{{\text{b}}}{{\text{a}}}} \right) $$

Divide Eq. (5) and Eq. (6), the pressure distribution in the air film can be obtained as

7 $$ {\text{p}} = {\text{p}}_{{\text{d}}} - \left( {{\text{p}}_{{\text{d}}} - {\text{p}}_{{\text{a}}} } \right)\frac{{\ln \left( {\frac{{\text{r}}}{{\text{a}}}} \right)}}{{\ln \left( {\frac{{\text{b}}}{{\text{a}}}} \right)}}\;{\text{or}}:\;{\text{q}}_{1,2 = } {\text{p}} = {\text{p}}_{{\text{d}}} - \left( {{\text{p}}_{{\text{d}}} - {\text{p}}_{{\text{a}}} } \right)\frac{{\ln \left( {\frac{{\text{r}}}{{\text{a}}}} \right)}}{{\ln \left( {\frac{{\text{b}}}{{\text{a}}}} \right)}} $$

The expressions of q1 and q2 are the same. For the air film flow field near the air supply hole, ${\text{p}}_{\text{d}}>{\text{p}}_{\text{a}}$; for the air film flow field near the vacuum hole, ${\text{p}}_{\text{d}}<{\text{p}}_{\text{a}}$.

### Calculation of the deformation of the glass substrate

**Figure 6:**
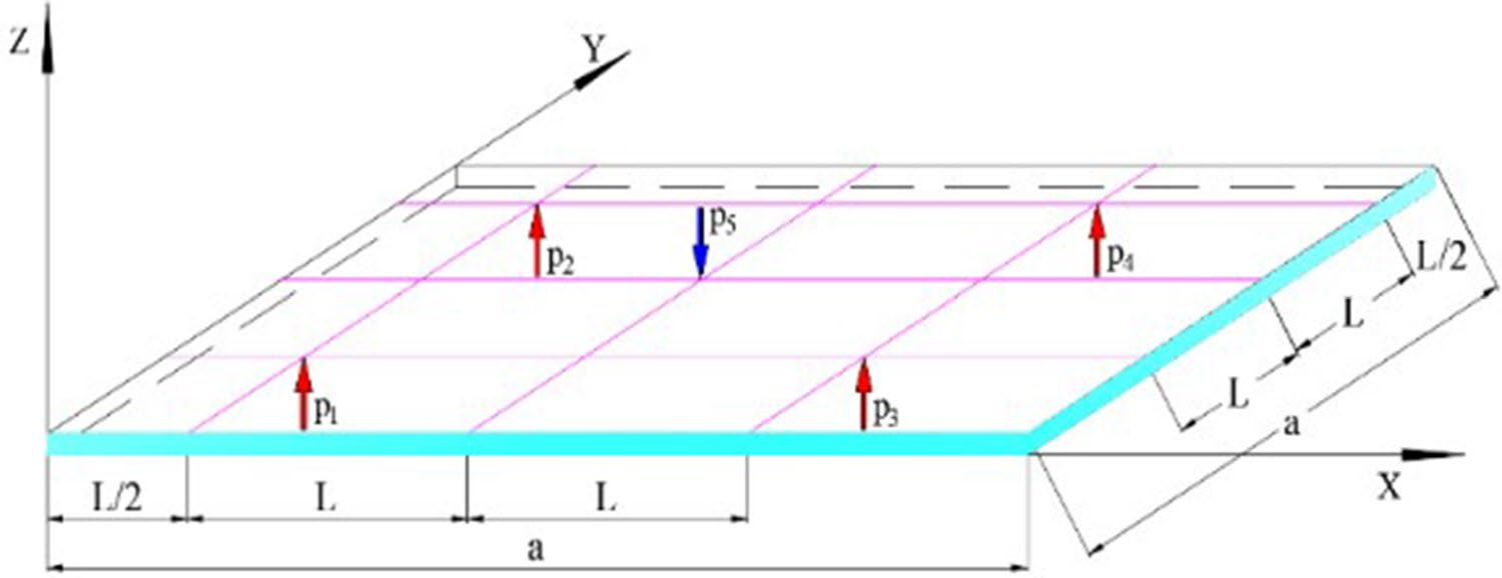
Glass substrate unit.

Take the glass substrate unit shown in Fig. 6 to analyze the deformation of the glass substrate. p1, p2, p3, p4 are air supply pressures. p5 is vacuum pressure. a, h, L respectively represent glass substrate unit length, its thickness, and the hole spacing of air-flotation plate. According to Timoshenko’s thin film theory^8^, the basic differential equation of the deformation of glass substrate is:

8 $$ \frac{{\partial^{4} \upomega }}{{\partial {\text{x}}^{4} }} + 2\frac{{\partial^{4} \upomega }}{{\partial {\text{x}}^{2} \partial {\text{y}}^{2} }} + \frac{{\partial^{4} \upomega }}{{\partial {\text{y}}^{4} }} = \frac{{{\text{q}}\left( {{\text{x}},{\text{y}}} \right)}}{{\text{D}}} $$

In Eq. (8), $\text{q}\left(\text{x},\text{y}\right)={\text{q}}_{1}-{\text{q}}_{2}-{\text{q}}_{3}$, ω is the deflection of the glass substrate. $\text{D}=\frac{({\text{Eh}}^{3})}{(12 (1-{\upgamma }^{2})}$ is the bending stiffness, E is the Young’s modulus, and V is the Poisson ratio.

**Figure 7:**
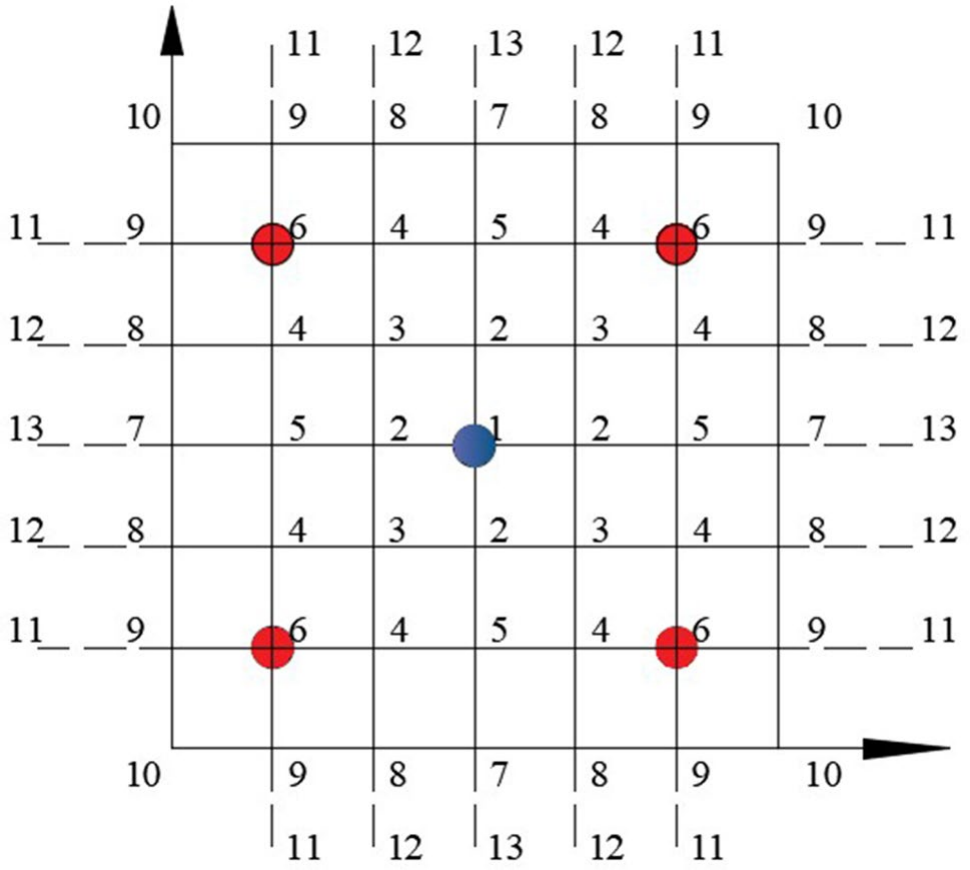
Computational mesh.

It is difficult to solve (8) with analytical method, and Eq. (8) can be solved with finite difference method. Considering that the deformation of the glass substrate is symmetrical, the mesh is shown in Fig. 7. Taking node 1 as an example, the fourth-order difference form can be obtained by simplifying the deflection function ω through Taylor expansion.

9 $$ \left( {\frac{{\partial^{4} \upomega }}{{\partial x^{4} }}} \right)_{1} = \left( {\frac{{\partial^{4} \upomega }}{{\partial y^{4} }}} \right)_{1} = \frac{1}{{n^{4} }}\left[ {6\upomega_{1} - 8\upomega_{2} + 2\upomega_{5} } \right],\;\,\left( {\frac{{\partial^{4} \upomega }}{{\partial x^{2} \partial y^{2} }}} \right)_{1} = \frac{1}{{n^{4} }}\left[ {4\upomega_{1} - 8\upomega_{2} + 4\upomega_{3} } \right] $$

In Eq. (9), n is the divided grid spacing.

Substitute Eq. (9) into Eq. (8) to obtain the deflection equation at node 1:

10 $$ 20\upomega_{1} - 32\upomega_{2} + 8\upomega_{3} + 4\upomega_{5} = \frac{{{\text{q}}_{1} {\text{n}}^{4} }}{{\text{D}}} $$

11 $$ \upomega = \frac{\partial \upomega }{{\partial {\text{x}}}} = 0 \left( {{\text{x}} = 0,\;{\text{x}} = {\text{a}}} \right), \;\;\;\upomega = \frac{\partial \upomega }{{\partial {\text{y}}}} = 0 \left( {{\text{y}} = 0,\;{\text{y}} = {\text{a}}} \right) $$

The boundary conditions in the form of difference are (take node 8 as an example):

12 $$ \upomega_{8} = 0 \;\;\;\upomega_{4} = \upomega_{12} $$

Thus, the equation describing the deformation of the glass substrate at six nodes is obtained:

$$   \begin{aligned}    & 20\omega _{1}  - 32\omega _{2}  + 8\omega _{3}  + 4\omega _{5}  = \frac{{q_{1} n^{4} }}{D} \\     &  - 8\omega _{1}  + 25\omega _{2}  - 16\omega _{3}  + 6\omega _{4}  - 8\omega _{5}  = \frac{{q_{2} n^{4} }}{D} \\     & 2\omega _{1}  - 16\omega _{2}  + 22\omega _{3}  - 16\omega _{4}  + 4\omega _{5}  + 2\omega _{6}  = \frac{{q_{3} n^{4} }}{D} \\     & 3\omega _{2}  - 8\omega _{3}  + 24\omega _{4}  - 8\omega _{5}  - 8\omega _{6}  = \frac{{q_{4} n^{4} }}{D} \\     & \omega _{1}  - 8\omega _{2}  + 4\omega _{3}  - 16\omega _{4}  + 21\omega _{5}  + 2\omega _{6}  = \frac{{q_{5} n^{4} }}{D} \\     & 2\omega _{3}  - 16\omega _{4}  + 2\omega _{5}  + 22\omega _{6}  = \frac{{q_{6} n^{4} }}{D} \\  \end{aligned}     $$

Or written as

13 $$ \left[ {\begin{array}{llllll}    {20} & { - 32} & 8 & 0 & 4 & 0  \\    { - 8} & {25} & { - 16} & 6 & { - 8} & 0  \\    2 & { - 16} & {22} & { - 16} & 4 & 2  \\    0 & 3 & { - 8} & {24} & { - 8} & { - 8}  \\    1 & { - 8} & 4 & { - 16} & {21} & 2  \\    0 & 0 & 2 & { - 16} & 2 & {22}  \\   \end{array} } \right]\left[ {\begin{array}{*{20}c}    {\omega _{1} }  \\    {\omega _{2} }  \\    {\omega _{3} }  \\    {\omega _{4} }  \\    {\omega _{5} }  \\    {\omega _{6} }  \\   \end{array} } \right] = \left[ {\begin{array}{*{20}c}    {\frac{{{\text{q}}_{1} {\text{n}}^{4} }}{{\text{D}}}}  \\    {\frac{{{\text{q}}_{2} {\text{n}}^{4} }}{{\text{D}}}}  \\    {\frac{{{\text{q}}_{3} {\text{n}}^{4} }}{{\text{D}}}}  \\    {\frac{{{\text{q}}_{4} {\text{n}}^{4} }}{{\text{D}}}}  \\    {\frac{{{\text{q}}_{5} {\text{n}}^{4} }}{{\text{D}}}}  \\    {\frac{{{\text{q}}_{6} {\text{n}}^{4} }}{{\text{D}}}}  \\   \end{array} } \right] $$

### Comparison between theoretical results and simulation results

Combined with examples, the deformation equation of the glass substrate is obtained with the employment of the theoretical method and finite element simulation. Example: Physical properties of glass substrate are shown in Table 1. Based on engineering experience, the pre-selected hole spacing is 20 mm (A model), 24 mm (B model) and 28 mm (C model) respectively. To ensure the accuracy of defect detection, the maximum deformation of the glass substrate is required to be within 1 mm. The supply pressure and vacuum pressure is 4 kPa and − 1 kPa respectively, both of which are gauge pressures.

**Table 1:** Physical properties of glass substrate.

Thickness	0.85 mm
Young’s modulus	70 Gpa
Poisson ratio	0.25
Density	2200 ${\text{kg}}/{\text{m}}^{3}$

In the process of solving the deformation of the glass substrate with the finite difference method, the model mesh is divided as shown in Table 2.

**Table 2:** Model mesh.

Model	Grid spacing (mm)	Number of grids	Number of nodes
A	3	20 × 20	55
B	4	18 × 18	45
C	6	14 × 14	28

In the finite element simulation process, the air film pressure is simulated first, and then the air film pressure is applied to the glass substrate to calculate the deformation. Table 3 presents the calculation results of the above two methods. It can be seen that the difference between the theoretical calculation and the finite element simulation is between 2 and 9.4%. Among them, the difference between model A is 2–4.6%, the difference between model B is 2.9–5.8%, and model C differs by 4.25–9.4%. Within a certain range, further grid refinement can improve the accuracy of the finite difference calculation.

**Table 3:** Comparisons of theoretical calculation and simulation.

Model	A		B		C	
	Glass center (mm)	Glass side (mm)	Glass center (mm)	Glass side (mm)	Glass center (mm)	Glass side (mm)
Theoretical calculation	0.45806	0.13893	0.95882	0.34286	1.8000	0.51710
Simulation	0.44892	0.13274	0.93157	0.32394	1.7266	0.47264

However, as the number of equations increases, the workload will also increase, so the balance between required accuracy and workload can be selected according to the accuracy requirement of the project.

The calculation results of the two methods manifest that when the hole spacing is 24 mm, the maximum deformation of the sheet is close to 1 mm; when the hole spacing is 28 mm, the maximum deformation of the sheet is close to 1.7266 mm, which does not meet the design requirements. Therefore, when considering the deformation of the glass substrate, the hole spacing should not exceed 24 mm.

## Experiments

**Figure 8:**
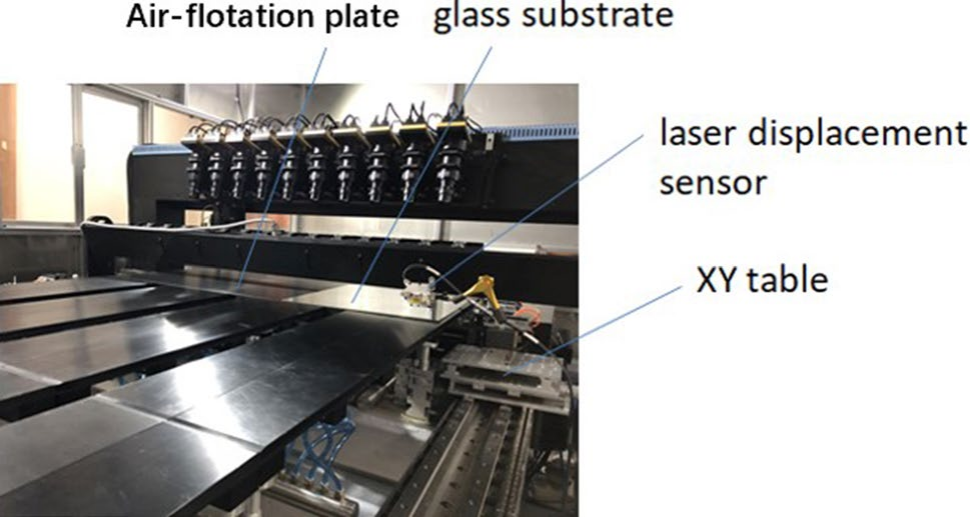
Optical detection prototype for glass substrate defect.

Based on the above theoretical analysis, air-flotation plates were designed with a hole spacing of 24 mm. As shown in Fig. 8, an experiment was carried out on an optical detection prototype to measure the maximum deformation of the glass substrate. The size of the glass substrate used in the experiment was 600 mm × 500 mm × 0.85 mm. A laser displacement sensor with the resolution of 0.1 μm is used for measurement. First measure the peak position, and then accurately move the probe to the trough position. The difference between the two points is the maximum deformation.

Repeat the measurement 5 times. The measurement results are shown in Table 4. The average maximum deformation of the glass substrate is 0.88 mm, which is in line with theoretical calculation expectations. The difference between the above experimental test results and the results of the finite difference method is less than 10%, indicating that the theoretical model meets the requirement of engineering design accuracy.

**Table 4:** Deformation measurements.

Number	Position of the wave peak (mm)	Position of the wave trough (mm)	Maximum Deformation (mm)
1	43.48	42.60	0.88
2	43.49	42.61	0.88
3	43.48	42.61	0.87
4	43.49	42.60	0.89
5	43.48	42.60	0.88

## Discussion

In this paper, the deformation of a large glass substrate in an orifice throttling air-flotation system is studied. According to the simulation graph of the pressure distribution of air film, an air load distribution model is established, and the expression of the deformation of the glass substrate is obtained. On this basis, the theoretical design method of designing nozzle pitch in air flotation system is given. The experimental results show that the error of the theoretical design method does not exceed 10%, which meets the engineering design requirements.

## Data Availability

The datasets generated during and/or analyzed during the current study are available from the corresponding author on reasonable request.
